# 早期非小细胞肺癌立体定向消融放疗现状

**DOI:** 10.3779/j.issn.1009-3419.2016.06.18

**Published:** 2016-06-20

**Authors:** 安辉 石, 广迎 朱

**Affiliations:** 100142 北京，北京大学肿瘤医院暨北京市肿瘤防治研究所放射治疗科 Department of Radiation Oncology, Key Laboratory of Carcinogenesis and Translational Research (Ministry of Education), Peking University Cancer Hospital and Institute, Beijing 100142, China

**Keywords:** 肺叶切除, 胸腔镜手术, 肺肿瘤, Lobectomy, Video-assisted thoracoscopic surgery, Lung neoplasms

## Abstract

目前比较立体定向消融放疗（stereotactic ablative radiotherapy, SABR）与手术治疗早期非小细胞肺癌（non-small cell lung cancer, NSCLC）的随机研究证据尚不多，高水平的循证医学证据更是缺乏。尽管STARS和ROSEL两项随机研究结果荟萃分析显示SABR较手术耐受性更好，生存不劣于手术，但是目前仍仅推荐拒绝手术或不可手术的早期NSCLC首选SABR，期待着正在进行的随机研究VALOR（Veterans Affairs Lung Cancer Surgery or Stereotactic Radiotherapy in the US）和SABRTooth（SABRTooth study in the United Kingdom）的结果。许多回顾性的研究和病例对照研究显示了SABR治疗的安全性和有效性（局部控制率达90%以上，5年生存率达70%），但是由于肿瘤分期定义、如何决定可否手术及手术患者采用手术方式（开胸或胸腔镜辅助）等不同，很难比较SABR和手术的优劣，尽管大部分结论是两种方法疗效相似，但难以成为循证医学证据，因此争论热点是哪一种方法更安全、创伤更小。本文将就以上争论热点进行述评。

## Abstract (en)

High level evidence from randomized studies comparing stereotactic ablative radiotherapy (SABR) to surgery is lacking. Although the results of pooled analysis of two randomized trials for STARS and ROSEL showed that SABR is better tolerated and might lead to better overall survival than surgery for operable clinical stage Ⅰ non-small cell lung cancer (NSCLC), SABR, however, is only recommended as a preferred treatment option for early stage NSCLC patients who cannot or will not undergo surgery. We, therefore, are waiting for the results of the ongoing randomized studies [Veterans affairs lung cancer surgery or stereotactic radiotherapy in the US (VALOR) and the SABRTooth study in the United Kingdom (SABRTooths)]. Many retrospective and case control studies showed that SABR is safe and effective (local control rate higher than 90%, 5 years survival rate reached 70%), but there are considerable variations in the definitions and staging of lung cancer, operability determination, and surgical approaches to operable lung cancer (open *vs* video-assisted). Therefore, it is difficult to compare the superiority of radiotherapy and surgery in the treatment of early staged lung cancer. Most studies demonstrated that the efficacy of the two modalities for early staged lung cancer is equivalent; however, due to the limited data, the conclusions from those studies are difficult to be evidence based. Therefore, the controversies will be focusing on the safety and invasiveness of the two treatment modalities. This article will review the ongoing debate in light of these goals.

肺癌仍是全球癌症死亡的首要原因，每年超过180万人被诊断为肺癌^[1]^。尽管以前的研究数据表明，肺癌患者诊断时只有25%为早期^[2]^，随着医学影像等相关检测技术的发展，诊断早期肺癌患者的比例有可能进一步提高。美国国立肺筛查试验（National Lung Screening Trial, NLST）^[3]^已经证实：与X线胸片筛查相比，采用低剂量螺旋计算机断层扫描（low-dose spiral computed tomography, LDCT）筛查肺癌高危人群可降低20%的肺癌死亡率，因此LDCT筛查可更早发现肺癌。临床分期为Ⅰ期的NSCLC标准治疗是肺叶切除术加纵隔淋巴结采样或清扫术，但是部分早期肺癌患者因高龄或伴随内科合并症而不能承受手术的风险，这部分患者采用常规分割放疗，3年总生存率为19%-30%^[4]^，而采用SABR，3年总生存率为55%-60%^[4]^，因此目前推荐拒绝手术或不可手术的早期NSCLC首选SABR。对于可手术的早期NSCLC，目前比较SABR与手术早期NSCLC的随机研究证据尚不多，高水平的循证医学证据更是缺乏，期待着正在进行的随机研究[VALOR（Veterans Affairs Lung Cancer Surgery or Stereotactic Radiotherapy in the US）和SABRTooth（the SABRTooth Study in the United Kingdom）等]结果。尽管许多回顾性的研究和病例对照研究显示了SABR治疗的安全性和有效性（局部控制率达90%以上，5年生存率达70%）^[5]^，但是由于肿瘤分期、可否手术的定义及手术患者采用手术方式（开胸或胸腔镜辅助）等不同，很难比较SABR和手术的优劣，尽管大部分结论是两种方法疗效相似，但难以成为循证医学证据，因此争论热点是哪一种方法更安全、创伤更小。本文将就以上争论热点进行述评。

## 1 治疗前分期评估

肺癌分期与生存密切相关，因此中国原发性肺癌诊疗规范^[6]^和美国胸科医师协会（American College of Chest Physicians, ACCP）^[2]^高度强调组织学诊断和准确分期检查的重要性。准确分期的关键是对纵隔淋巴结进行全面评估。尽管评价SABR和手术的前瞻性研究没有要求每例患者必须获得组织学诊断和/或纵隔淋巴结活检，但是患者均经历了正电子发射型计算机断层显像（positron emission computed tomography, PET）/CT分期，既往多中心研究显示PET/CT判断N0与N1/N2具有较高的阴性预测值（91%）^[7]^，因此对于拒绝手术或不可手术T1-2的患者，进行SABR治疗具有重要意义。当然，不可否认前瞻性的随机研究显示了手术在NSCLC淋巴结评估的重要性^[8]^，美国外科医生协会肿瘤学研究组（American College of Surgery Oncology Group, ACOSOG）z0030试验数据显示525例早期肺癌患者行淋巴结清扫术后有16%的隐匿的N1或N2^[8]^。即使是非常小的肿瘤（1 cm）也有7%的患者伴有隐匿性淋巴结转移^[9]^。因此对于可疑转移患者采用侵入性的方法是必要的。随着电磁导航支气管镜、超声内镜引导下的经支气管针吸活检（endobronchial ultrasound-guided transbronchial needle aspiration, EBUS-TBNA）、超声内镜（endoscopic ultrasonography, EUS）、CT引导下穿刺活检等先进技术的应用，更应该强调对疑似肺癌患者的准确诊断和分期。

## 2 不可手术的早期NSCLC的SABR治疗

20世纪50年代，SABR技术已经在颅内肿瘤治疗中使用^[10]^。颅外第一次使用SABR技术是在20世纪90年代，SABR技术在早期NSCLC应用的关键特征是：图像引导技术、呼吸门控技术、二维成像技术及金属标记放置在肿瘤内部或周围实时跟踪技术的融合。计划靶体积（planning target volume, PTV）是基于肿瘤体积（gross tumor volume, GTV）考虑肿瘤运动及摆位误差外放形成。SABR技术较常规放疗技术发生放射性肺炎和纤维化肺炎的几率更低。2001年Uematsu等^[11]^首先报道了SABR技术治疗经病理证实的早期NSCLC的临床结果，该研究包括50例T1和T2的患者，29例内科原因不能手术，21例可手术但拒绝手术的患者，内科不能手术患者3年生存率66%，21例可手术患者3年生存率86%。50例患者，仅局部复发3例，仅淋巴结复发0例，仅远地转移5例，远地转移伴淋巴结复发2例。Timmerman等^[12]^2005年设计并完成SABR技术治疗早期NSCLC的Ⅰ期剂量递增临床试验，该研究入组47例不可手术病理诊断明确的Ⅰa期或Ib期肺癌患者，局部复发率为21%，治疗毒性主要是放射性肺炎、心包炎、气管塌陷，最大的耐受剂量为66 Gy/3次。因为SABR技术较常规放疗在治疗早期肺癌方面的优势，美国肿瘤放射治疗协作组织（Radiation Therapy Oncology Group, RTOG）在2004年-2006年开展SABR技术治疗病理证实的早期NSCLC的Ⅱ期临床研究（RTOG 0236）^[13]^，55例患者入组，其中80%为T1a期，中央型肺癌由于并发症发生率高被排除在外^[14]^，是否可手术由胸内科和胸外科医生决定。局部失败定义为在计划靶体积边缘1 cm内（大体肿瘤体积边缘1.5 cm内）出现肿瘤复发，局部区域失败包括原发部位、同一肺叶、肺门或纵隔。远地转移定义为局部区域失败之外的部位出现转移。结果显示，3年总生存率和无病生存率分别为55%和48.3%，局部区域失败和远地转移率分别为12.8%和20%。22%的患者在3个月内发生了3级或更高的不良反应，包括肺功能下降、低血钙或肺炎（未报道肋骨骨折和胸壁疼痛）。由于RTOG 0236研究没有治疗相关的死亡报道，从而SABR技术得到迅速广泛应用。随后一些研究报道了SABR技术治疗早期NSCLC 3年的生存结果^[15-17]^，详见表 1。

**1:** SABR技术治疗不可手术早期非小细胞肺癌3年的生存结果 3-year survival results of inoperable early-stage NSCLC by SABR

Author	Year	*n* (evaluable)	Stage (*n*)	Dose	Follow-up (mo)	OS (mo)	LCR (%) (yr)	OS (%) (yr)	Toxicity
Timmerman R^[17]^	2010	59 (55)	T1 (44)	54 Gy/3 f/1.5 w-2 w	34.4	48.1	97.6 (3)	55.8 (3)	Lever Ⅲ (*n*=7)
			T2 (11)						Lever Ⅳ (*n*=2)
Ricardi U^[16]^	2010	62	T1 (43)	45 Gy/3 f/1 w	28	NR	87.8 (3)	57.1 (3)	Lever Ⅲ pneumonitis (3%)
			T2 (19)						Rib fracture (2%)
Baumann P^[15]^	2008	57	T1 (40)	54 Gy/3 f/1.5 w-2 w	35	NR	90 (3)	60 (3)	Level Ⅲ chest pain (*n*=2)
			T2 (17)						Fibrosis (*n*=2)
									Rib fracture (*n*=1)
f: fractions; w: week; Md: median; OS: overall survival; NR: not reported; LCR: local control rate; SABR: stereotactic ablative radiotherapy; NSCLC: non-small cell lung cancer.									

## 3 可手术的早期NSCLC的SABR治疗

表 2把可手术早期NSCLC的SABR治疗结果与手术结果进行了比较^[8, 18-20]^，结果显示与手术疗效相似。随着越来越多深受鼓舞的回顾性分析结果报道，两项前瞻性的随机研究相继开展，STARS研究由美国、中国、法国多个中心（7个中心入组患者）参与，ROSEL研究主要来自于荷兰十个中心（4个中心入组患者），但因入组缓慢等原因，STARS和ROSEL研究相继关闭（STARS研究共入组31例，ROSEL研究共入组21例）。由于两项研究相似的入组标准，Chang等^[21]^对STARS和ROSEL研究结果进行荟萃分析，SABR组与手术组两组患者基本情况无统计学差异，两项研究中手术均采用肺叶切除术加纵隔淋巴结采样或清扫术，外周型病灶放疗剂量均为54 Gy/3次，中央型病灶放疗剂量分别为50 Gy/4次（STARS研究）和60 Gy/5次（ROSEL研究）。结果显示，SABR组与手术组中位随访时间分别为40.2个月和35.4个月，3年生存率分别为95%个79%（*P*=0.037），3年无复发生存率分别为86%和80%（*P*=0.54），研究结果更支持SABR治疗，因此作者推测SABR可作为早期NSCLC手术治疗之外的一种选择，仍需要更高水平的循证医学证据支持。而未来两项前瞻性的随机研究（美国退伍军人医院的VALOR研究和英国的SABRTooth研究）将会回答哪些患者将从SABR治疗中获益。

**2:** 可手术的早期非小细胞肺癌SABR治疗与手术比较 Comparison of SABR versus. surgery for operable early-stage NSCLC

Author	Year	*n*	Stage (*n*)	Doses	Follow-up (mo)	OS (mo)	LCR (%) (yr)	OS (%) (yr)	Toxicity
Lagerwaard FJ^[18]^	2012	177	T1 (106)	60 Gy/3 f-8 f/ 1.5 w-2 w	31.5	61.5	93% (3)	84.7 (3)	Level Ⅲ pneumonitis (3%)
			T2 (71)						Rib fracture (2%)
Onishi H^[19]^	2011	87	T1 (65)	45 Gy-72.5 Gy/3 f-10 f/0.6 w-2 w	55	NR	92% (3) 73% (5)	72 (Ⅰa) (5)	Level Ⅲ (*n*=1)
			T2 (22)					62 (Ⅰb) (5)	
Nagata Y^[20]^	2015	64	T1 (65)	48 Gy/4 f/4 d-8 d	45.4	NR	NR	76 (3)	Level Ⅲ chest pain (*n*=1)
									Dyspnea (*n*=2)
									Pneumonitis (*n*=2)
Darling GE^[8]^	2011	1, 023	T1 (578)	Surgery	78	97.2 (biopsy)	NR	72 (5)	NR
			T2 (440)			102.0 (dissection)		55 (5)	

## 4 讨论

尽管手术是早期肺癌的标准治疗，但随着非手术治疗方法的快速发展，如SABR技术，已在可手术早期NSCLC治疗中崭露头角，在拥有令人满意的疗效同时，治疗相关的并发症较低，但最终仍需大宗前瞻性的随机研究进一步证实。目前，SABR技术已经成为拒绝手术或不可手术的早期NSCLC首选治疗方式。随着电磁导航支气管镜、EBUS、EUS、CT引导下穿刺活检等先进技术的发展，而且在强调肺癌多学科团队治疗的时代，治疗前应尽可能给患者取得病理明确诊断，除了参考肺癌病理，尚需参考肿瘤的遗传信息（分子测序），以便更好的指导患者后续的靶向治疗。相信未来前瞻性的随机研究将会除外没有病理的患者，会利用现有手段更精准的分期，结果将会更有说服力。

## 5 结论

手术切除仍然是早期NSCLC的标准治疗。对于拒绝手术或不可手术的早期NSCLC，SABR是一种安全有效的治疗选择。患者可否手术应该由胸外科团队评估，在大宗前瞻性的随机研究进一步证实之前，除了临床研究之外，应慎重对可手术早期NSCLC开展SABR治疗。
